# Epidemiology and control of bovine ephemeral fever

**DOI:** 10.1186/s13567-015-0262-4

**Published:** 2015-10-28

**Authors:** Peter J. Walker, Eyal Klement

**Affiliations:** CSIRO Health and Biosecurity, Australian Animal Health Laboratory, 5 Portarlington Road, Geelong, VIC 3220 Australia; Koret School of Veterinary Medicine, The Hebrew University, 76100 Rehovot, Israel

## Abstract

Bovine ephemeral fever (or 3-day sickness) is an acute febrile illness of cattle and water buffaloes. Caused by an arthropod-borne rhabdovirus, bovine ephemeral fever virus (BEFV), the disease occurs seasonally over a vast expanse of the globe encompassing much of Africa, the Middle East, Asia and Australia. Although mortality rates are typically low, infection prevalence and morbidity rates during outbreaks are often very high, causing serious economic impacts through loss of milk production, poor cattle condition at sale and loss of traction power at harvest. There are also significant impacts on trade to regions in which the disease does not occur, including the Americas and most of Europe. In recent years, unusually severe outbreaks of bovine ephemeral fever have been reported from several regions in Asia and the Middle East, with mortality rates through disease or culling in excess of 10–20%. There are also concerns that, like other vector-borne diseases of livestock, the geographic distribution of bovine ephemeral fever could expand into regions that have historically been free of the disease. Here, we review current knowledge of the virus, including its molecular and antigenic structure, and the epidemiology of the disease across its entire geographic range. We also discuss the effectiveness of vaccination and other strategies to prevent or control infection.

**Electronic supplementary material:**

The online version of this article (doi:10.1186/s13567-015-0262-4) contains supplementary material, which is available to authorized users.

## Introduction

**Figure 1:**
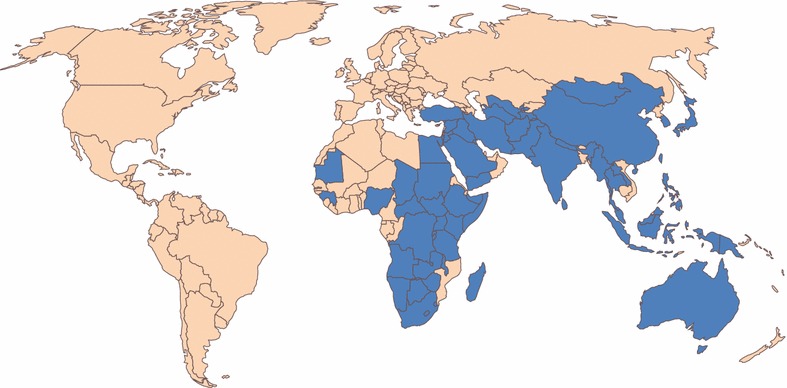
**Countries in which BEF is known to occur or from which the disease has been reported historically (shown in blue).** The extent of BEFV distribution is not necessarily country-wide (as shown) and may include neighbouring countries from which there are no known formal reports of disease (not shown). The distribution may also vary seasonally and from year to year.

Bovine ephemeral fever virus (BEFV) an arthropod-borne rhabdovirus which is classified as the type species of the genus *Ephemerovirus*. It causes an acute febrile illness of cattle and water buffalo known as bovine ephemeral fever (BEF) or various other local names such as 3-day sickness, bovine enzootic fever, bovine influenza or stiffseitke. It occurs over a vast expanse of the globe from the southern tip of Africa to the Nile River Delta, across the Middle East through South and South-East Asia, into northern and eastern Australia, and throughout most of China, extending into Taiwan, the Korean Peninsula and southern Japan (Figure 1). BEFV does not occur in the islands of the Pacific, Europe (other than in the western regions of Turkey) or in the Americas where, for quarantine purposes, it is considered as an important exotic pathogen. Infection may be clinically unapparent or result in mild to severe clinical signs including a bi-phasic fever, salivation, ocular and nasal discharge, recumbency, muscle stiffness, lameness and anorexia. Sternal and lateral recumbency in cattle with clinical BEF are shown in Additional file 1. Usually, the disease is characterised by rapid onset and rapid recovery, lasting only 1–3 days, but there are reports of prolonged paralysis and ataxia in some animals following the acute phase of infection. The most severe cases can result in mortality which may be due to exposure, starvation or pneumonia, but little is currently known about the direct cause of death. Morbidity rates can be very high (approaching 100%) and mortality rates are typically low (<1%). However, in recent years there have been reports from several countries of alarmingly high case-fatality rates, sometimes exceeding 20% [1–3]. The economic impacts of BEF can be considerable and are due primarily to cessation of lactation in dairy cattle, loss of condition in beef cattle and the immobilisation of water buffalo used for draught power [4–7]. A recent study has estimated an average net loss of 175.9 kg milk per cow affected by BEF [7]. BEF also impacts on trade in live cattle from infected zones and there is some evidence that the risks of inter-continental spread of BEFV through animal transport or vector translocation may be increasing [8].

## Molecular and antigenic characteristics of BEFV

### Molecular structure

**Figure 2:**
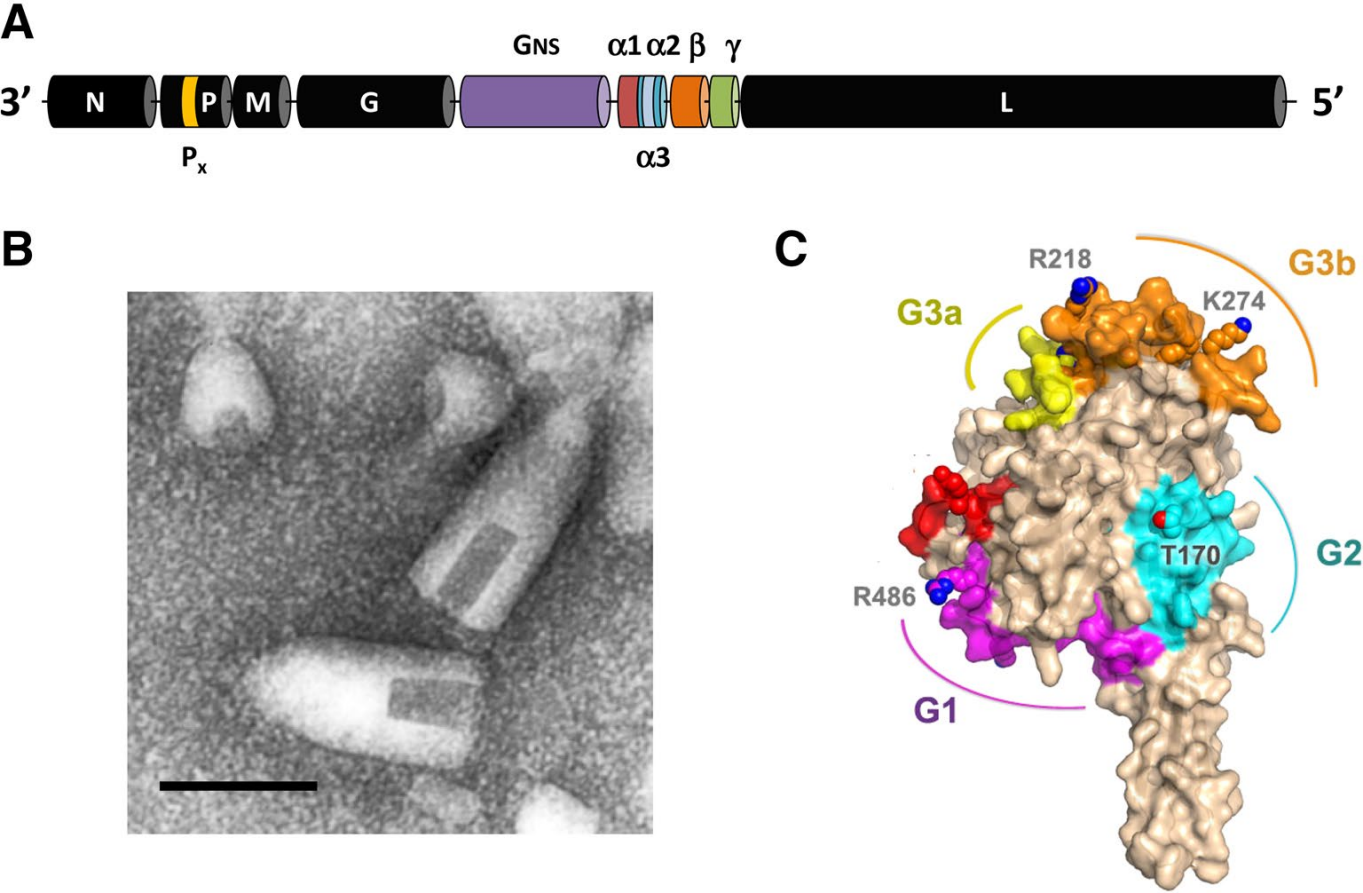
**Structure and morphology of BEFV.** **A** Structural organization of the 14.9 kb BEFV genome shown as arranged in negative sense. Structural protein genes (N, P, M, G and L) are shown in black and the various accessory genes are coloured. **B** Transmission electron micrograph showing BEFV virions and defective-interfering (DI) particles. Scale bar 100 nm. Reproduced from Walker [7] with permission from Springer-Verlag. **C** Structural model of a monomeric subunit of the BEFV G protein derived by homology modelling using the pre-fusion form of the VSV G protein as a template. The model illustrates the three major neutralization sites (G1, G2 and G3a/b) and amino acid residues shown to be under positive selection in Australia [18]. Adapted with permission from the American Society for Microbiology.

BEFV displays typical rhabdovirus bullet-shaped morphology (Figure 2), although virions (~185 nm × ~75 nm) appear to be more tapered at one end than the rounded forms that are observed for vesicular stomatitis virus (VSV) or rabies virus (RABV) [9]. Helical nucleocapsids comprise the negative-sense, single-stranded RNA genome tightly associated with the 52 kDa nucleoprotein (N) which, together with the 43 kDa phosphoprotein (P) and the large multi-functional enzyme (L) form a ribonucleoprotein complex [10]. Nucleocapsids are encased in the 29 kDa matrix protein (M) and a lipid envelope through which an 81 kDa class 1 transmembrane glycoprotein (G) protrudes to form surface projections [9]. Defective-interfering particles with truncated cone-shaped morphology are commonly present in purified virus preparations (Figure 2).

The 14.9 kb BEFV genome is much larger and more complex than those of VSV or RABV, possibly explaining the subtle difference in virion morphology (Figure 2). In addition to the five canonical rhabdovirus structural protein genes, the BEFV genome features a ~3.4 kb region between the G and L genes containing multiple additional open reading frames (ORFs) [11]. Thus, the genome contains a total of 10 long open reading frames (ORFs) arranged in the order 3′-N-P-M-G-[G_NS_-α1-α2-β-γ]-L-5′ (in negative sense). The G_NS_ gene encodes a 90 kDa class 1 transmembrane glycoprotein that is structurally related to the G protein and appears to have been generated by gene duplication [12]. The G_NS_ protein is expressed in infected cells but is not incorporated into virions and, unlike the G protein, does not appear to be fusogenic at low pH [13, 14]. Its function is presently unknown. The α1 gene encodes a 10.5 kDa transmembrane protein that is expressed in infected cells and has the structural and functional properties of a viroporin [15]. BEFV α1 protein localises to the Golgi complex and has been shown to bind importin β1 and importin 7, suggesting that it may also have a role in modulating nuclear trafficking pathways [15]. The functions of proteins encoded in the α2, β and γ ORFs are presently unknown. However, it has been observed that expression of the β and γ ORFs is selectively suppressed by mutation during adaptation to cell culture, suggesting that their role is associated with infection in vivo (P.J. Walker, unpublished data). Short alternative ORFs also occur in different frames within the P and α2 ORFs but it is not known if they are expressed as functional proteins.

### Antigenic structure

The BEFV G protein is the target of virus-neutralising antibodies [16, 17]. The G protein shares structural homology with the G proteins of other animal rhabdoviruses [18, 19], including the VSV G protein for which the crystal structure has been resolved. Three major neutralisation sites (G1–G3) have been defined by using BEFV G protein-specific monoclonal antibodies (MAbs) in competition ELISAs [17]. The sites have been confirmed by selection of neutralisation escape mutants [20] and physically mapped to the G protein structure by sequence analysis of the mutants [18, 21]. A fourth site (G4) has also been defined by a MAb-selected neutralisation escape mutant but it has not yet been mapped physically [20]. G1 is a linear neutralisation site (Y^487^-K^503^) that is located at the end of the trimerisation domain (DII) just before the C-terminal stalk of the G protein [18, 21]. It comprises two minimal B cell epitopes that map to each end of the site [21]. G2 is a conformational site which is located at the base of the fusion domain (DIV) adjacent to a disulphide bridge (C^172^–C^182^) that forms a loop containing a predicted *N*-glycosylation site. G3 is the major conformational site in the BEFV G protein, occupying most of the base of the pleckstrin homology (PH) domain (DIII). It comprises amino acids from three linear regions of the G protein that are aligned by the fold. It includes two sub-sites (G3a and G3b) defined by either partial or complete inhibition of MAb binding in competition ELISAs [18, 21]. Each of these antigenic sites is predicted to be exposed at the surface of the G protein (Figure 2). Both G2 and G3 are predicted to be available to neutralising antibodies in the pre-fusion form of the G protein [18]. Site G2 is on the lateral face of the trimer. Site G3 is at the distal end of the spike and corresponds to the major conformational sites of VSV and RABV which are thought to be involved in receptor-binding. Site G1 is predicted to face the viral membrane in both the pre-fusion and post-fusion forms of the trimer and may be accessible to antibodies only during a transitional monomeric phase [18].

The BEFV N protein is also immunogenic in cattle and in mice. It does not induce virus-neutralising antibodies or a protective response [22, 23] but it does induce a T cell proliferative response in cattle [22]. All 12 available BEFV N protein MAbs are non-neutralising and have been mapped to non-conformational sites in the C-terminal half of the protein. Two of these MAbs have been shown to cross-react in immunoblots with the rabies virus N protein [23] and this may explain weak cross-reactions detected in indirect immunofluorescence tests between certain lyssaviruses and ephemeroviruses [24]. Monoclonal antibodies have also been generated to the BEFV M protein. They are all non-neutralising and appear to bind to non-conformational sites but have not been physically mapped to the protein [17].

### Antigenic variation

BEFV is considered to exist as a single serotype worldwide. Various neutralisation tests conducted using isolates from Australia, China, Japan, Kenya, Nigeria and South Africa have demonstrated strong antigenic cross-reactions [25–29]. There is also anecdotal evidence that vaccines developed in several countries using BEFV strains isolated more than 40 years ago remain effective against currently circulating strains and that vaccines developed against a strain of the virus from one region are effective against viruses currently circulating in other regions of the world. Nevertheless, homologous neutralisation titres are typically higher than heterologous titres amongst viruses isolated at different times or from different geographic regions [1, 27, 28].

Variations have also been detected in the major neutralisation sites of the G protein. A study of 66 Australian BEFV isolates collected between 1956 and 1992 has indicated that, whilst all isolates were neutralised by MAbs representing antigenic sites G1, G2 and G4, variations have occurred in some epitopes within the major conformational site G3, allowing the identification of four antigenic sub-types [18, 20]. Isolates assigned to subtype I were neutralised by MAbs (generated against the 1968 isolate BB7721) representing all four antigenic sites; subtype II isolates lack an epitope in site G3a; subtype III isolates lack an epitope in site G3b; and subtype IV isolates lack both the G2a and G3b epitopes. Interestingly, whilst variations in the site G3b epitope showed no temporal or geographic association, the G3a epitope was found to be present in all viruses isolated prior to 1973–74 and absent from all viruses isolated since that time [18]. Sequence analysis of the isolates indicated that variations in site G3a were associated with a conservative substitution at amino acid 218 (R218K) and that variations in the site G3b epitope were primarily associated with various substitutions or deletions at amino acid 215 (Figure 2). Variations were also observed in other amino acids in regions that had been mapped previously to sites G1, G2 or G3 but they did not affect the neutralisation phenotypes of epitopes targeted by the MAbs used in the study [18]. Analyses of the G protein sequences of BEFV isolates from Japan and Taiwan, mainland China and the Middle East have also revealed variations in amino acids in regions that correspond to antigenic sites G1, G2 and G3 [3, 30–32]. However, the MAb-neutralisation phenotypes of these isolates have not been determined.

## Ecology of BEF infection

### Vertebrate host range

Clinical BEF has been reported only in cattle and water buffalo (*Bubalus bubalis*). Although cattle are considered to be more susceptible to disease [33, 34], mild clinical signs have been reproduced experimentally in water buffalo [35] and severe disease has been reported in the field [36]. There is also evidence of infection and clinical BEF in yak (*Bos grunniens*) in China and India [37, 38]. Serological studies have detected BEFV antibodies in a wide range of wild ungulates. In surveys conducted in Kenya, Tanzania, Zimbabwe and South Africa, BEFV-neutralising antibodies have been detected in African buffalo (*Syncerus caffer*), waterbuck (*Kobus ellipsiprymnus*) wildebeest (*Connochaetes taurinus, Connochaetes gnou*), hartebeest (*Alcelaphus buselaphus*), topi (*Damaliscus korrigum*), tsessebe (*Damaliscus lunatus*), blesbok (*Damaliscus dorcas phillipsi*), springbok (*Antidorcus marsupialis*), impala (*Aepycerus melampus*), sable antelope (*Hippotragus niger*), eland (*Taurotargus oryx*), kudu (*Tragelaphus strepsiceros*), bushbuck (*Tragelaphus scriptus*) and giraffe (*Giraffa camelopardalis*) [39–42]. In other African, countries exposure to BEFV infection has been reported in lechwe (*Kobus leche*), elephant (*Loxodonta africana*), warthog (*Phacochoerus aethiopicus*), oryx (*Oryx beisa*), hippopotamus (*Hippopotamus amphibius*) and gazelle (*Gazella granti*) [41]. In some cases, the prevalence of BEFV antibodies in African wildlife was quite high (over 60% of animals tested), suggesting they may serve as natural reservoirs of infection in which the virus cycles during inter-epizootic periods [39, 42]. BEFV-neutralising antibodies have also been detected in Persian fallow deer (*Dama d. mesopotamica*) and gazelle (*Gazella g. gazella*) in Israel [43], pigs (*Sus scrofa*) in Korea [44], and red deer (*Cervus elaphus*), Rusa deer (*C. timorensis*) and Chital deer (*Axis axis*) in Australia [5, 45, 46]. There is also evidence of infection in camels (*Camelus dromedaries*) in Egypt and Somalia [47] and an ephemeral fever-like illness known locally as “Lahaw-Gaal” has been reported to affect camels in Somalia and north-eastern Kenya [48]. Generally, the low prevalence of BEFV antibody in these species and their quite small populations relative to cattle and water buffalo suggests that they may have little importance in the overall ecology of infection outside of Africa.

Sheep have also been infected experimentally with BEFV but did not develop clinical signs other than pyrexia and attempts to isolate the virus failed [49, 50]. However, some sheep developed BEFV-neutralising antibodies and mild haematological changes, and clinical disease was observed in susceptible steers inoculated with leukocytes collected from the infected sheep 3–4 days after infection. Although BEFV-neutralising antibodies have been reported in sheep and goats in Taiwan [51], several other serological studies conducted in BEFV-enzootic regions have failed to find evidence of infection in sheep [43, 45, 52]. Factors such as low levels of viraemia and the feeding preference of vectors may limit the role of sheep in the natural BEFV transmission cycle.

### Vector-borne transmission

A large body of evidence suggests that BEFV is transmitted by haematophagous insects. Its geographical distribution is mostly in tropical, subtropical and warm temperate regions and the pattern of disease is seasonal with outbreaks occurring from late spring to autumn [6]. It has also been shown that experimental transmission of infection requires intravenous injection of infected blood and there is no transmission by direct contact with infected animals or fomites [53, 54]. Attempts to transmit BEFV mechanically from various insects were also unsuccessful [53]. BEFV has been isolated from several potential haematophagous vector species including biting midges and mosquitoes. The virus has been isolated from *Culicoides imicola* and *C. coarctus* in Zimbabwe [55], from a mixed pool of biting midges in Kenya, comprising *C. kingi*, *C. nivosis*, *C. bedfordi* and *C. pallidipennis* [29], and from *C. puncticollis* in Turkmenistan [56]. In Australia, there have been isolations from *C. brevitarsis* [57], *Anopheles bancroftii* mosquitoes and a mixed pool of mosquitoes that included *Culex, Uranotaenia* and *Aedes* spp. [58, 59]. These isolations have been from insects that were not recently blood-engorged.

Attempts to demonstrate vector competence for BEFV in mosquitoes and biting midges have met with limited success. Artificial membrane-feeding experiments showed no evidence of BEFV replication in *Aedes vigilax* but replication to some extent was detected in *Culex annulirostris* 10 days after feeding [60]. In other experiments, BEFV was recovered from three of 23 *Cx. annulirostris* 12 days after feeding on a blood-virus mixture [59]. It has also been reported that up to 70% of *Cx. annulirostris* inoculated intrathoracically with BEFV were found by in vitro capillary tube feeding to be excreting virus 7 days after incubation at 26 °C [61]. In contrast, BEFV was recovered from only one of 526 *C. brevitarsis* 10 days after feeding on a mixture of sucrose and infected mouse brain. In a large study conducted in South Africa, biting midges (primarily *C. imicola* and *C. bolitinos*) collected in the field were fed blood mixed with Australian and African strains of BEFV. Although BEFV was detected in 18.9% of the midges assayed immediately after feeding, none of the >4000 midges surviving at 10 days post-feeding were found to be infected [62].

Various other factors also appear to implicate mosquitoes as the principal vectors of BEFV. The observation that direct intravenous injection is required to initiate experimental BEFV infection in cattle suggests that capillary feeders (mosquitoes) rather than pool feeders (midges) would be required for efficient transmission [61]. This is supported by evidence that experimental BEFV infection is confined primarily to the blood and bone marrow with no evidence of infection in the peripheral lymph system [63]. It has also been observed that the geographic distribution of BEFV in Australia extends beyond that of the most widely distributed midge species (*C. brevitarsis*) but is similar to the distribution of *Cx. annulirostris* mosquitoes [64]. The epidemiological pattern in Australia, in which outbreaks commonly follow heavy rainfall, also suggests an association with the emergence of large populations of mosquitoes breeding in shallow ground pools [63]. However, the distribution of BEFV in Kenya has been reported to extend beyond the zones in which mosquitoes are abundant, and its appearance in locations from which other mosquito-borne diseases (such as Rift Valley fever) have not been isolated, may suggest transmission by midges [65]. Further work is required to better define the vectors of BEFV, including vector competence studies to demonstrate transmission following the extrinsic incubation period by vectors fed on infected cattle. It is possible that several species of midges and mosquitoes could serve as vectors when seasonally abundant.

## History, distribution and epizootiology of BEF

### Global distribution

**Figure 3:**
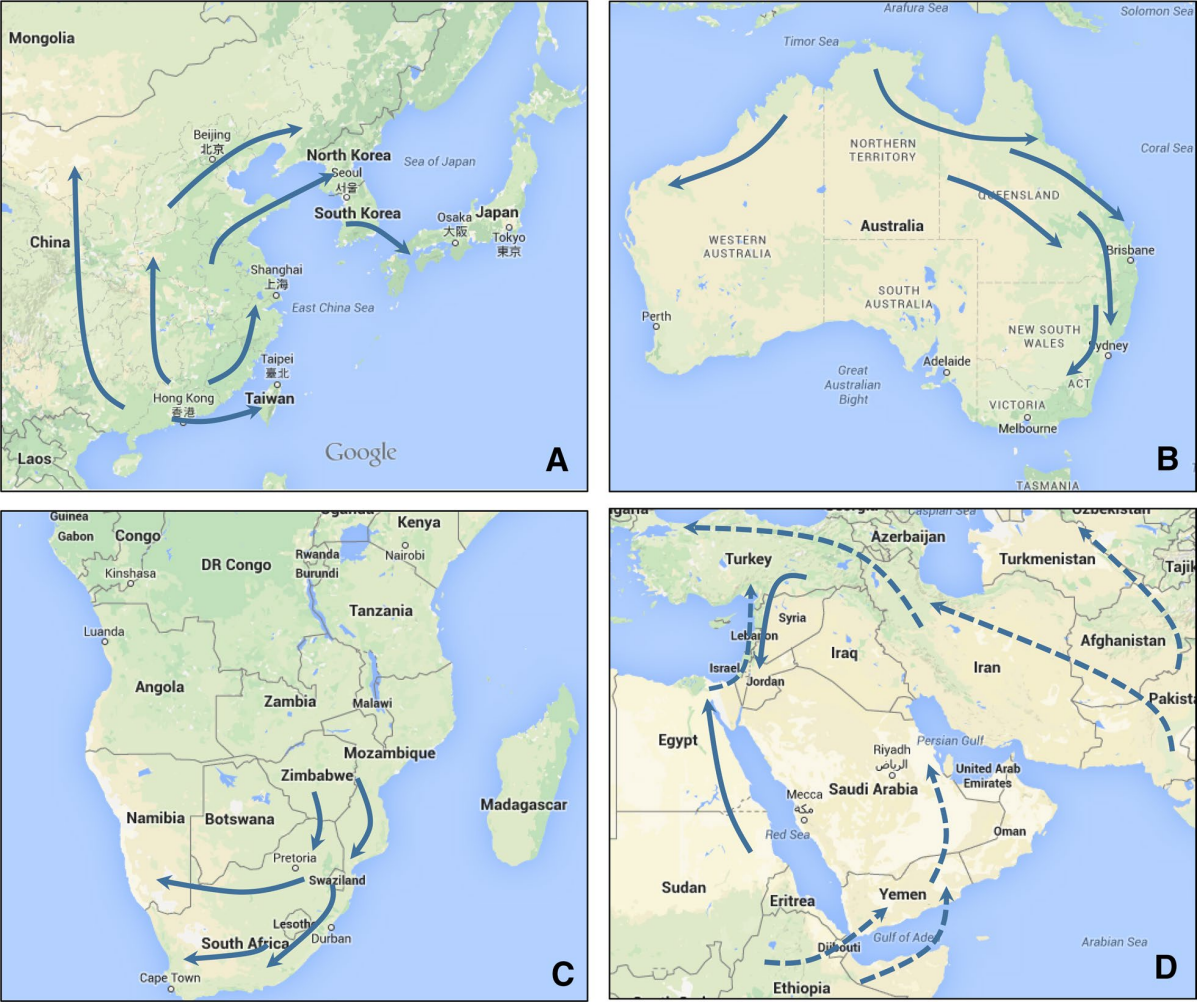
**Likely general directions of seasonal spread of BEFV.** **A** East Asia. **B** Australia. **C** Southern Africa. **D** Middle East. Epizootics appear to emerge in the spring or early summer from enzootic foci in tropical regions and extend northwards or southwards through late summer and autumn. Pathways shown in East Asia and Australia are based on historical records and recent observations of epizootics, supported by molecular epidemiological studies. Pathways in Africa are based only on historical accounts. Pathways in the Middle East are less clear and may be complex with potential for epizootics to originate in either East Africa or West Asia. Dashed arrows indicate possible pathways in this region.

**Figure 4:**
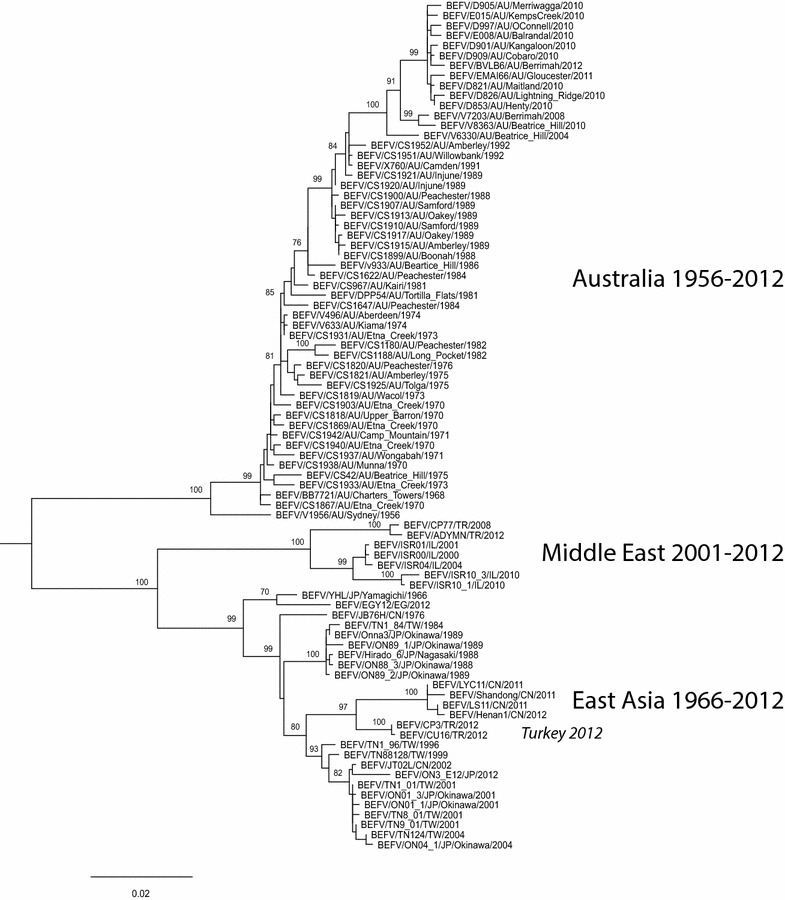
**Phylogenetic tree of nucleotide sequences of the G protein ectodomains (1527 nt) of BEFV isolates showing the known global genetic diversity of the viruses.** The tree was generated from a MUSCLE alignment of the sequences using the maximum likelihood method, the GTR + gamma model for nucleotide substitution and SPR branch swapping. Bootstrap analysis was conducted using 100 replicates. Genbank accession numbers for all sequences used in the analysis are provided in Additional file 2.

Bovine ephemeral fever has been described in many tropical and sub-tropical regions around the world (Figure 1). It is enzootic and seasonally epizootic in Australia, Asia, Africa and the Middle East, usually not extending beyond a zone limited by the latitudes if 38°N to 36°S [6, 66]. Epizootics commonly move northwards or southwards in a wave-like fashion, commencing in tropical enzootic foci in the spring or early summer and subsiding in autumn (Figure 3). Although BEFV is believed to exist as a single serotype, phylogenetic studies using G gene ectodomain sequences have shown that the available BEFV isolates cluster geographically into 3 lineages: East Asia, Australia and the Middle East [2, 3, 8, 18, 32] (Figure 4). The G gene is a useful genotyping marker as it displays reliable alignment with adequate sequence variation to obtain precision and resolution, and the absence of genetic recombination suggests it is likely to be is representative of the entire genome. However, genotype analyses to date have been based on limited sampling from most regions and there are no sequences available for BEFV isolates from Africa or countries in Central, South or South-East Asia.

### Australia

The disease is enzootic in Australia. It was first reported in 1936 when an outbreak occurred 450 km south of Darwin in the Northern Territory [67]. The remoteness of this location suggests that BEFV was likely present but undetected prior to the outbreak which occurred 60 years after the establishment of large cattle holdings in the north of the continent. Prior to the late 1970s, BEF occurred primarily as large epizootics that swept southwards in a wave-like fashion out of the northern tropical zone into sub-tropical and warm temperate regions of Western Australia, Queensland and New South Wales, occasionally reaching northern Victoria. Such epizootics occurred in 1936–37, 1955–56, 1967–68, and then in succession in 1970–71, 1972–74 and 1974–76 corresponding to periods of unusually high annual rainfall associated with the La Niña phase of the Southern Oscillation Index [6, 68–70]. Since that time, the epidemiological pattern changed from an advancing north-south wave to annual outbreaks during the summer and autumn months over a wide area of northern and eastern Australia [71]. Regular monitoring of sentinel herds has indicated that there are marked differences in seasonality between different geographic regions [72]. Sero-conversions occur perennially in the northern tropical zone of the Northern Territory but seasonality increases gradually with increasing latitude in eastern Australia where transmission may cease during the winter months, particularly during periods of prolonged drought. BEFV sero-conversions also occur annually in the Kimberley region in the far north of Western Australia and are detected regularly in the Pilbara region approximately 1000 km to the south-west.

Molecular epidemiological studies using G gene ectodomain sequences have shown that all BEFV strains isolated in Australia since 1956 have a single common ancestor indicating that, unless earlier lineages have become extinct, the virus has entered Australia on only a single occasion [18]. The phylogenetic data also indicate that BEFV is evolving as a single clade across the continent at the relatively high evolutionary rate of ~10^−3^ nucleotide substitutions/site/year [18]. This suggests that most adaptive evolution occurs in the perennial enzootic focus in the north where strains are continually selected for optimum fitness and regularly move south with the onset of summer and autumn rains, displacing the scattered remnants of enzootic infection. There is also evidence that the evolution of BEFV in Australia is being driven by cross-reactive neutralising antibody to Kimberley virus [18] which has a similar geographic distribution and ecology to BEFV but has not been associated with clinical disease (see below).

### South and South-East Asia

Although the epidemiology is poorly defined, there is evidence of BEFV infection in an arc of countries in South and South-East Asia extending from the Indian sub-continent and Sri Lanka, eastwards to the Indonesian Archipelago and northwards to the Philippines. An ephemeral fever resembling BEF was first reported in Pakistan (then Punjab) in 1919 as a disease of cattle known locally as “Vil” [73]. It was noted that the disease occurred most commonly during the rainy season (July to October) and simultaneous outbreaks at widely separated locations suggested that it was insect-borne. The disease was also reported in Tamil Nadu in the south-east of India in 1924 [74]. More recently, BEF has been reported in cattle and water buffalo in Uttar Pradesh in the central north [75], Gujarat in the west [36] and Himachal Pradesh in the far north [76]. In Uttar Pradesh during 1973–1975, BEF was reported to occur from April to October with the highest incidence in July and August when the weather is hot and humid. Disease occurred more frequently in *Bos taurus*/*Bos indicus* cross-bred animals than in local breeds and the highest incidence occurred in older cattle [75]. In Nepal, BEF also occurs during the hot, humid months of June to October and is considered to be sporadic in some regions and enzootic in others [77]. In Sri Lanka, BEF occurs primarily from June to December and appears to follow periods of high rainfall [78, 79].

BEF was first reported in Indonesia in 1919 when an outbreak occurred in dairy cattle in West Java. The next reported epizootic occurred from 1928 to 1931 on the east coast of Sumatra [80]. Subsequent outbreaks were reported in East Java in 1978, where cases persisted at least until 1985 with sometimes quite high mortality rates, and in Kalimantan in 1991. A serological survey conducted in 1979 detected a high prevalence (78.9%) of BEFV-neutralising antibodies in cattle from Java and Bali [81]. Serological surveys conducted between 1987 and 1990 also detected a relatively high prevalence of BEFV-neutralising antibodies in sentinel cattle across much of the archipelago including Sumatra, Java, Bali, Timor, Kalimantan, Sulawesi and West Papua [80, 82]. Sero-conversions were seasonal, occurring primarily during the wet season from December to June. A BEF epizootic also occurred in Papua New Guinea in 1959 but subsequent serological surveys in sentinel herds between 1969 and 1975 failed to detect evidence of infection [68].

Less information is available from other countries in the region. A BEF-like illness was reported in water buffalo in the Philippines in 1936 and passed experimentally in water buffalo and cattle, resulting in a brief, non-fatal febrile illness [83]. BEF was also reported in the Philippines in 1975–1976 when it affected cattle and water buffalo in 44 provinces in 12 regions, with a case-fatality rate of 5% in water buffalo [84]. There were also local news reports in the Philippines of the disease in January 2011. A serological survey conducted in southern Thailand in 1982 revealed BEFV-specific neutralizing antibodies in 70% of cattle tested from 11 provinces and 47.5% of water buffalo sampled from four provinces [85]. The disease has also been reported to occur sporadically in cattle and water buffalo in Malaysia, especially following heavy rain after period of dry weather [86]. Mortality rates were reported to be low but higher in water buffalo than in cattle. There are also unconfirmed historical reports (1964–1969) of BEF in Laos and Singapore [66].

There have been no confirmed isolations of BEFV from South or South-East Asia and no molecular epidemiological data are available at this time.

### China and East Asia

BEF is enzootic in mainland China [37]. It was first reported in Jiangsu Province (north of Shanghai) in 1934 when the disease was called bovine influenza [87]. Since 1955, BEF has been recorded in all provinces except Xinjiang and Qinghai in the west, Heilongjiang in the far north-east and the densely populated areas of Tianjin and Hebei [87]. In Guangdong Province in the south-east, outbreaks occur almost every year and large epizootics occur in the south every 2 years, usually commencing in June or July and lasting until November. Approximately every 4 years, the large epizootics move further northward in a wave-like fashion, affecting provinces such as Henan and Anhui in August and September [87]. An outbreak in Jilin Province in 1991 remains the most northerly latitude (44°N) at which the disease has ever been recorded [88]. A serological survey conducted in cattle, water buffalo and yak during 2011–2014 detected BEFV-neutralising antibody in 26 of 28 provinces across the country, including Heilongjiang in the far north-east and Xinjiang in the west [37]. Sero-prevalence varied by location and year with highest prevalence (81%) in cattle from Shaanxi Province in 2012. Although mortality rates due to BEF in China have been typically low (case fatality rates <2%), severe disease with case-fatality rates of 17–18% were reported during three epizootics in Henan Province in 2004, 2005 and 2011 [3]. Phylogenetic analysis based on G gene sequences of isolates from the 2011 epizootic showed that they clustered with other BEFV isolates from mainland China, Taiwan and Japan [3, 18]. The source of this strain and the reasons for the high mortality rates is presently not known. There have been no similar large epizootics in Henan since 2011 and case-fatality rates in other provinces have not exceeded 2% (Prof. Hong Yin, Lanzhou Veterinary Research Institute, personal communication).

Epizootics also occur periodically in Taiwan, Japan and the Korean Peninsula. In Japan, the disease has been known since large epizootics, then called bovine influenza, were recorded in 1889 and 1893 [89]. Similar epizootics were recorded in 1906–1908, 1929 and 1949–1950 when the disease was renamed bovine epizootic fever and recognised as having the characteristics of BEF [90]. There were frequent epizootics in the 1950s and 1960s but no major outbreaks have occurred since the introduction of vaccination in 1973 [91]. Recent outbreaks have been sporadic and confined to islands of Okinawa Prefecture in the southernmost region of Japan [30, 92]. In South Korea, BEFV sero-conversions were detected during 2009–2012 in sentinel cattle from all provinces except heavily populated Incheon and Busan [93]. The sero-prevalence and distribution varied from year to year with highest prevalence (35.7%) recorded in Daegu Province in 2011. It has been shown that BEF epizootics in the Korean Peninsula and nearby Fukuoka Prefecture in Japan follow a similar pattern [91]. Major epizootics occurred simultaneously in 1955, 1958, 1966, 1988 and 1991 whilst smaller sporadic outbreaks occurred independently in one or other of the countries in other years. It has been proposed that low-level jet stream winds allow the displacement of BEFV-infected vectors across the South China Sea and the Sea of Japan [91, 94, 95].

Although the disease called bovine influenza had long been known to occur in Taiwan, the first confirmed BEF epizootic was in 1967 when an outbreak occurred in the Kaoshiung District in the south of the island [96]. Since that time, nine epizootics have been reported; those occurring in 1983–1984, 1988–1990 and 1996 have been described as sweeping epizootics whilst those occurring in 1999, 2001, 2002, 2004, 2007 and 2012–2013 were focal or multi-focal [1, 31, 97, 98]. In most cases the first outbreaks were reported in southern Taiwan but the 1996 epizootic commenced in central Taiwan and the 2002 outbreak commenced in eastern Taiwan. There has been a clear trend towards more frequent epizootics since 1967 with falling morbidity rates but increasing case-fatality rates. Mortalities due to disease and culling exceeded 10% in most epizootics since 1989–1990 and reached 50% in 2002 [1]. The falling morbidity rate has been attributed to the introduction of a vaccine in 1984. The increased mortality rate has been associated with a change in the nature of the disease from an acute self-limiting infection to a more chronic condition in which symptomatic treatment with anti-inflammatory drugs and calcium borogluconate are ineffective [97]. During the epizootic in 1996, there was evidence that co-infection with Ibaraki virus (family *Reoviridae*, genus *Orbivirus*) may have occurred in some cattle, contributing to the prolonged nature of the disease and poor prognosis [96]. A study of 23 BEF outbreaks which occurred in Taiwan during the period 2001–2013 suggested a possible association between low neutralising antibody titers and the occurrence of outbreaks. However, statistical significance for this association was not demonstrated [98].

Phylogenetic analysis using G gene ectodomain sequences has shown that isolates from China and East Asia collected during the period from 1966 to 2012 form a single clade that is distinct from the Australia and Middle East clades [3, 18, 30, 31, 98] (Figure 4). There are three major sub-clades within the China/East Asia clade comprising isolates that cluster chronologically. The first comprises isolates from Taiwan and Japan collected during the 1984 and 1988–89 epizootics; the second comprises isolates from Taiwan, Japan and China collected between 1996 and 2012; the third comprises 2011–2012 isolates from China. A 1976 isolate from China and the 1966 vaccine strain from Japan are at the deepest ancestral nodes of the China/East Asia clade. The phylogeny suggests that BEFV in China/East Asia is evolving largely as a single clade and indicates a close epidemiological association between viruses circulating across the region.

### Central Asia

There is little published information readily available on the occurrence of BEF in Central Asia. Chunikhin and Alekseev [99] referred to the presence of BEFV in the former Soviet Union. Sporadic outbreaks have been reported in the Amu Darya, Pyandzh and Vahsh Valleys in Tajikistan and Uzbekistan, and two BEFV isolates have been reported from midges (*Culicoides puncticollis*) collected in 1980 from camels in Turkmenistan [56]. There is also a report of a BEFV isolate, apparently obtained from Mongolia in 1993, which has been used for vaccine production in response to disease in territories bordering Russia, including Central Asia and Mongolia [100]. A BEF outbreak was also reported in Tajikistan in 2002, affecting the Moskva, Pyandzh and Parkhar districts bordering Afghanistan [101]. Epidemiologically, this vast region could link the Middle East to China and South Asia and so viral sequence data and further information on the status of the disease would be highly valuable.

### Middle East

There are detailed data on the occurrence of BEF in Egypt, Israel, Turkey and Saudi Arabia. Serological data collected in 2011 has also revealed exposure to BEFV in Jordan, primarily in the Jordan Valley (Almajali, personal communication). There are also reports of BEF in Iran [102, 103] and the disease was recorded in Kuwait, Yemen, Iraq and Syria prior to the cessation of formal reporting to the OIE in 1970 [66].

In Egypt, BEF was known as “dengue fever” of cattle in the late 19th century but the first detailed report was of an epizootic in 1909 that commenced at Aswan, travelled down the Nile Valley to Cairo and spread across the Delta to the coast [104]. Subsequent outbreaks affecting hundreds of cattle occurred in 1915 and 1919–1920. More recently, the disease was reported during the summers of 1990–1991, 2000–2001 and 2004–2005 [34, 105]. A major epizootic in 1990–1991 affected cattle throughout the country, moving along the Nile Valley from Upper Egypt in the summer of 1990 to the eastern part of the Delta in the autumn [34]. In 1991, the disease affected 250,000 imported cattle and a smaller number of indigenous cattle and water buffalo all along the Nile Valley, in the Delta and at several oases west of the Nile. Morbidity rates were reported to vary from 20 to 90% and mortality rates in imported cattle were 1.5–3.0%. The source of the virus was believed to have been by aerial displacement of vectors from regions to the south or the east [34]. Molecular epidemiological studies of BEFV in Egypt have been confined to date to a single report that a virus isolated in 2005 was very closely related to a 2004 isolate from Taiwan [8]. It was suggested that the virus may have been imported through the cattle trade from China to the Middle East. However, as this is based on a short sequence of the G protein (140 amino acids) that is identical to the Taiwanese sequence, the result requires confirmation. A full-length G protein sequence of a 2012 Egyptian BEFV isolate which has been deposited in Genbank (KJ729108) is most closely related to the 1966 Japanese vaccine strain (Figure 4).

BEF was first reported in Israel in 1931 [106]. Until 1990, it occurred at irregular, long intervals, with the last outbreak occurring in 1951 [107]. However, this pattern has changed in recent years with increasingly frequent outbreaks in 1990–1991, 1999–2001, 2004, 2008, 2009 and 2010 [8, 107, 108]. The first descriptions of BEF in Saudi Arabia and Turkey appeared much later. In Turkey, the first reported BEF outbreak occurred in 1985 in the central, south and south-eastern parts of Anatolia, and this was followed by outbreaks in 1999, 2003, 2008 and 2012 [32]. Although most outbreaks occurred in Anatolia with predominance in the southern part of the country (2, 8, 32), sero-prevalence of 2.5–15.3% has been reported in the western provinces of European Turkey [109]. This is the first report of BEF in Europe.

The first unconfirmed outbreaks of BEF in Saudi Arabia occurred in 1980 [110]. Subsequent disease outbreaks in 1990–1991 and 1996 were confirmed serologically [111, 112] but a survey conducted from 1993 to 1995 revealed no evidence of BEFV antibodies in 910 cattle sampled from sentinel herds at six locations across the country [113]. Although this indicated that the disease had not remained enzootic since the 1990–1991 outbreak, another epizootic occurred during 1996, affecting both exotic and local breeds in the eastern region of the country. The disease was confirmed serologically and by virus isolation [114] and it was suggested that the virus may have been introduced in arthropod vectors transported on prevailing south-westerly winds from Africa [113]. There have been no further reports of outbreaks in Saudi Arabia or other countries in the Arabian Peninsula.

BEF outbreaks in the Middle East usually commence during the spring (May) or autumn (September) when the rains are ceasing and ambient temperatures rise. In Israel, the most severe outbreaks usually occur in the Jordan Valley were the climate is either Mediterranean or semi-arid while in Saudi Arabia, which is mostly arid, the outbreaks usually occur at oases in which large populations of vectors can emerge [112]. There has been increased frequency of epizootics in several countries in the region during the past 20 years and several observations suggest there may be a connection between the outbreaks which have occurred simultaneously in Israel, Egypt and Saudi Arabia in 1990, in Turkey, Israel and Egypt in 1999–2000, in Egypt and Israel in 2004, and in Turkey and Israel in 2008. Spread of infection between these sites may have occurred either by wind-borne dispersal of infected vectors or by transportation of infected cattle. As serological surveys conducted between epidemics have indicated the absence of infection [8, 113], the intermittent reintroduction of the virus from neighboring enzootic countries in Asia and Africa appears to be the most likely source of epizootics.

### Sub-Saharan Africa

BEF is enzootic and seasonally epizootic in Africa. The first recorded epizootic commenced in north-western Zimbabwe (then Rhodesia) in November 1906. From central Zimbabwe, the disease advanced southwards to reach Natal and Transvaal by March 1907 and, by the end of 1907, it was reported near Port Elizabeth in Eastern Cape Province where it remained with intervals of quiescence [50]. Indigenous residents claimed that the disease had occurred in Zimbabwe 25 years previously and there was a report of a similar disease in native cattle in Central Africa dating to approximately 1867 [50, 115]. Although transmission by birds associated with locust plagues was originally proposed [50], biting midges were subsequently implicated by analogy with bluetongue disease [115]. Major epizootics were subsequently reported in South Africa in 1949, 1953–1955, 1966–68, 1974–74 and 1981–84 [116]. The epizootic of 1953–1955 was particularly severe with mortality rates approaching 30% in some herds [117]. BEF is now reported to occur regularly in South Africa from the northern border to the southern coast. Clinical disease occurs less frequently in some districts and the severity and extent of outbreaks may vary from year to year. The disease typically appears in the late summer, but can occur earlier in the winter rainfall region of the Western Cape Province [118]. It has also been reported to occur annually in Namibia where it occurs later than in South Africa with outbreaks sometimes extending into the winter [54].

The disease has also been reported to occur in Sudan, Kenya, Uganda and Tanzania [66, 119]. BEF was first recognised in Kenya in 1913 and reproduced experimentally in cattle [65]. It has since occurred at intervals, usually associated with years of greater than average rainfall when Rift Valley fever was also prevalent, although an outbreak was recorded in a region adjacent to saline lakes in the absence of recent rain [39, 120]. Epizootics occur in all parts of the country and can vary with respect to morbidity rates and severity of clinical signs. In inter-epizootic periods, seroconversions have been observed in cattle and wild ruminants the absence of clinical disease [39, 120]. Antibodies to BEFV have also been reported in camels from Somalia [47]. BEFV has been isolated from cattle and biting midges in Kenya and from cattle in Nigeria [26, 29]. In Nigeria, the disease has been known to herdsmen for many years, occurring regularly at the beginning of the wet season [26].

BEF has also been reported historically to occur sporadically or seasonally in many other African countries including Chad, Mauritania, Guinea, Central African Republic, Ethiopia, Rwanda, Burundi, Democratic Republic of Congo, Zambia, Malawi, Angola, Botswana, Lesotho, Swaziland and Madagascar [66].

### Wind-borne dispersal

A significant body of evidence suggests that BEFV dispersal within geographic regions occurs by wind-borne displacement of vectors. Spread of BEF in Australia during the 1968–1969 epizootic was in accordance with a combination of monsoonal influence and an intense low pressure system that developed in inland Queensland [69]. This resulted in the wave-like spread of BEF cases along an eventual front of 800 km, moving progressively from the Northern Territory to northern Victoria, 3000 km to the south. This spread was in the opposite direction to the movement of most livestock [70]. In 2008, the rapid progression of BEF cases from north-western New South Wales to central and southern parts of the state was preceded by the southward movement of a low-pressure system [121]. Both epizootics were preceded by heavy rainfall that followed a prolonged drought, stimulating the emergence of large vector populations in the affected areas.

Wind-borne dispersal of vectors has also been implicated in BEF epizootics in East Asia and the Middle East. BEFV incursion into Japan and Korea has been associated with the wind direction of a low-level jet stream from China [91, 94] and this is supported by the genetic similarity between viruses isolated during disease outbreaks in each country [30]. A close genetic relationship has also been observed for BEFV isolates from an outbreak in Turkey during 2008 and an outbreak in Israel a few months later. Forward and backward wind trajectory analysis revealed that air parcels originating in a highly affected region of southern Turkey reached Israel 9 days prior to detection of the index case. As there was no known cattle trade between Israel and Turkey prior to the outbreak, no evidence for serological exposure of cattle and only minor exposure of wild animals to BEFV in the inter-epidemic period (2004–2008), and greater genetic similarity between the 2008 Israel and Turkish isolates than to a virus circulated in Israel during 2000, wind-borne displacement of vectors from Turkey was considered to be the most likely source of the epizootic strain [8, 43].

### Translocation through the live animal trade

The phylogenetic clustering of BEFV isolates according to geographic regions suggests that long distance (inter-continental) dispersal of BEFV by animal transport has been rare historically. However, several studies have shown that recent BEFV isolates from Egypt (2005) and Turkey (2012) cluster phylogenetically with isolates from the East Asian clade rather than the Middle East clade [8, 18, 32]. It has been reported that about 5000 cattle were exported from China to the Middle East 1 year prior to the 2005 outbreak in Egypt. Although no cattle were imported from East Asia to Turkey prior to the 2012 outbreak, transportation may have occurred to neighboring countries. It is also possible that the East Asian lineage virus in Turkey may have been imported by transportation of cattle via Africa. A large outbreak of lumpy skin disease, which is usually confined almost exclusively to Africa, also occurred in northern Israel and Lebanon during 2012 and has been attributed to the legal or illegal trade in cattle from Africa to the Middle East. The phylogenetic data certainly suggests that the risk of inter-continental BEFV translocation through the live animal trade may be increasing.

## Other ephemeroviruses

Several other viruses that have been isolated from cattle or biting insects are antigenically related to BEFV, some of which have been classified as members of the genus *Ephemerovirus*. From a clinical perspective, the most significant of these is kotonkan virus (KOTV) which was isolated from biting midges (*Culicoides* spp.) in Nigeria in 1967 [122]. Seroconversion to KOTV neutralising antibody has been associated with an ephemeral fever-like illness in cattle in Nigeria [122] and mild signs of the disease have been observed following experiment infection of calves with a mouse brain-adapted strain of the virus [123]. Based primarily on antigenic cross-reactions with Mokola virus in complement-fixation and indirect immunofluorescence tests [24, 122], KOTV was originally classified as a lyssavirus but sequence analysis has clearly established its classification as a species (*Kotonkan virus*) in the genus *Ephemerovirus* [124]. Other established ephemerovirus species include *Berrimah virus* (BRMV), *Adelaide River virus* (ARV) and *Obodhiang virus* (OBOV). BRMV was isolated in 1981 from a healthy sentinel steer in the Northern Territory of Australia [125]. Antigenically, it is the most closely related ephemerovirus to BEFV, cross-reacting weakly in virus-neutralisation tests [124, 125]. Although there is evidence of widespread BRMV antibody in cattle in Australia, it has never been associated with clinical disease. Neutralising antibodies to BRMV have also been detected in cattle in China [87] and in cattle, water buffalo, sheep and goats in Indonesia [82]. ARV was also isolated from a healthy sentinel steer in the Northern Territory in 1981 and has no known association with disease [126]. It is most closely related antigenically and phylogenetically to OBOV which was isolated in 1963 from mosquitoes (*Mansonia uniformis*) in Sudan with which it cross-reacts weakly in virus-neutralisation tests [124]. Although little is known about the ecology or geographic distribution of these viruses, antibodies to ARV have also been detected in cattle in China and in water buffalo and goats in Indonesia [82].

Kimberley virus (KIMV), Malakal virus (MALV), Koolpinyah virus (KOOLV), Yata virus (YATV) and Puchong virus (PUCV) have not yet been classified formally but are likely to be assigned to the genus *Ephemerovirus* based on serological and phylogenetic relationships, and similarities in genome organisations and host/vector associations. KIMV was first isolated from mosquitoes (*Culex annulirostris*) collected in Western Australia in 1973 [127] and then subsequently on several occasions from biting midges (*Culicoides brevitarsis*) and healthy sentinel cattle in the Northern Territory and Queensland [45, 57, 128]. KIMV is indistinguishable in virus neutralisation tests from MALV which was isolated from mosquitoes (*Mansonia uniformis*) in Sudan in 1963, and these are now considered to be variants of the same virus species [129]. KIMV antibodies have been detected in cattle in China [130] and in cattle, water buffalo, goats and horses in Indonesia [82]. KOOLV was isolated in 1985 and 1986 from healthy sentinel cattle in the Northern Territory and shown to cross-react in virus-neutralisation tests with KOTV. At the time of the isolations, there was evidence of sero-conversion to KOOLV antibody in other cattle at the same site and in sheep infected experimentally with the virus. Subsequent sequence analysis of the KOOLV genome has established that it is indeed closely related to KOTV with a similar genome organisation and high levels of amino acid sequence identity between cognate proteins [131]. YATV was isolated in 1969 from mosquitoes (*Mansonia uniformis*) collected in the Central African Republic. Recent studies have established that YATV clusters phylogenetically with the ephemeroviruses and shares a similar genome organization [131]. PUCV was isolated in 1965 in Malaysia, also from *Mansonia uniformis* mosquitoes and was subsequently shown to cross-react in complement-fixation tests with MALV (strain SudAr-1169) and in indirect immunofluorescence tests with several other ephemeroviruses [24]. Recent sequence analysis has confirmed that PUCV is indeed an ephemerovirus, most closely related to KIMV (P.J. Walker and K.R. Blasdell, unpublished data). Further studies are required to better define the vectors, host range and prevalence, geographic distribution and pathogenicity of these poorly characterised ephemeroviruses.

## Control and treatment of BEF

### Protective immunity

Natural BEFV infection has been reported to result in durable immunity [53]. There have been observations of multiple episodes of clinical ephemeral fever in the same cattle [5, 115, 132] but it is not known if other ephemeroviruses may have been responsible for the disease. A strong neutralising antibody response follows natural or experimental BEFV infection, developing by the third day of clinical disease with titres increasing during recovery [133, 134]. It has been reported that specific neutralising antibodies last for at least 422 days following natural BEFV infection and that previously infected animals resist challenge for at least 2 years [135]. There are conflicting reports on the role of neutralising antibodies in protection against the disease. Tzipori and Spradbrow [136] observed that cattle developing a neutralising antibody response following vaccination with mouse-brain-adapted virus were not consistently resistant to challenge. Della-Porta and Snowdon [137] found no correlation between the magnitude of the neutralising antibody response to vaccination and protection, and suggested that cell-mediated responses may also be required. However, others have observed a correlation between BEFV-specific neutralising antibody titer and protection and effective protection has been demonstrated using purified preparations of the G protein split from virions [16, 31, 138]. Inclusion of N protein in the purified G protein vaccine, although stimulating a T-lymphocyte proliferative response, did not improve protective efficacy [22]. Colostral antibody has also been shown to protect cattle against BEFV infection [139] and neutralising G protein monoclonal antibodies injected intraperitoneally protect suckling mice from paralysis and death [17]. Therefore, it appears that the G protein delivered in an appropriately folded form and with a suitable adjuvant is sufficient to induce protective immunity. A key role for neutralising antibodies in protection is also consistent with the short incubation period, rapid onset of disease and rapid recovery that coincides with the first appearance of neutralising antibody [133]. However, it is plausible that cell-mediated immunity is also involved in protection, particularly for the longer term sequelae that occur in some animals.

There is also evidence that innate immunity is involved in both the immune response to infection and the pathology of disease. In an elegant experiment, Young and Spradbrow [140] challenged calves with BEFV after depletion of neutrophils with a specific anti-bovine neutrophil serum of equine origin. Although becoming viraemic, the calves did not develop clinical signs and no BEFV-neutralising antibodies were detected. However, virus challenge following restoration of neutrophils resulted in viraema, clinical signs and a specific neutralising antibody response. It appears, therefore, that neutrophils are important in the induction of clinical signs and in the development of the humoral immune response. This is consistent with evidence that the pathology associated with BEFV infection is primarily due to vascular permeability and the cytokine storm resulting from the associated inflammatory response [132].

### Vaccines

Four types of BEF vaccine have been developed to date: (1) live-attenuated vaccines; (2) inactivated vaccines; (3) sub-unit G protein-based vaccines; and (4) recombinant vaccines. Live-attenuated, inactivated and subunit vaccines are being used in the field. Vaccination has been adopted to varying extents in Australia, South Africa, Namibia, Japan, South Korea, Taiwan, mainland China, the Philippines, Turkey, Israel, Kuwait, Oman, Bahrain, Saudi Arabia and Egypt. The vaccines differ in the seed virus from which they are prepared, the method of attenuation or inactivation, and the adjuvant formulation.

Live-attenuated vaccines have been prepared by serial passage of BEFV in suckling mice and/or in cell cultures, including baby hamster kidney (BHK-21), hamster lung (HmLu-1) or African green monkey kidney (Vero) cells [6, 141, 142]. Many of these live-attenuated vaccines have been administered with aluminium hydroxide or Freund’s incomplete adjuvant and require volumes of up to 12 mL/dose. A live-attenuated vaccine employing Freund’s incomplete adjuvant has been adopted for commercial use in South Africa [143]. Vanselow et al. [138] have reported that two 1 mL doses of an attenuated BEF vaccine mixed with Quil A (a purified saponin derivative) induced higher neutralising antibody titers than when using aluminium hydroxide or dextran sulphate as adjuvants. The field effectiveness of this vaccine was reported to be 90% (2.9% morbidity in vaccinated animals as compared to 24.9% in non-vaccinated cattle) and similar effectiveness was observed in a herd naturally infected 12 months after vaccination [144]. Although it has been claimed that Quil A, which is mixed with the vaccine in the field shortly before administration, inactivates at least 99.99% of the virus [138], this effect may be due to aggregation of virus particles rather than inactivation of infectivity [6]. The live-attenuated Quil A vaccine has been adopted for commercial use in Australia [144].

Inactivated BEF vaccines have been prepared by treatment with formalin [142], β-propriolactone [142, 145], binary ethyleneimine [31] or ultraviolet light [142]. Heat-inactivation of the virus resulted in failure to induce a neutralising antibody response after vaccination [136]. Most early inactivated vaccines used either aluminium gel or Freund’s incomplete adjuvant [142]. A formalin-inactivated, aluminium phosphate gel-adsorbed vaccine developed in Japan was shown to elicit a strong antibody response after two doses but immunity waned rapidly and neutralising antibody was no longer detected in most animals 4 months after vaccination [142]. More recently, inactivated vaccines have used water-in-oil-in-water adjuvant [31, 145]. Such a vaccine developed in Israel was shown to elicit a stronger and longer lasting neutralising antibody response after two vaccinations and showed a significant booster effect 9 months after the second vaccination. No safety issues have been reported with this vaccine and no effect was observed on milk production in vaccinated cattle [145]. An oil emulsion vaccine developed and tested in Taiwan showed 100% protection against experimental challenge performed 1 month after a single vaccination [31]. The Israeli vaccine showed 50% effectiveness in protection from natural challenge after three vaccinations but failed to prevent disease when administrated only twice [7]. This is consistent with previous challenge studies which found that inactivated vaccines elicit protection only after three doses [137].

Inaba et al. [146] found that consecutive vaccinations with live-attenuated virus followed by inactivated (killed) virus resulted in a stronger and more durable neutralising antibody response than vaccination with live-attenuated vaccine alone or with two doses of the inactivated vaccine. No abortions or foetus damage were observed and there was no reduction in milk production following vaccination with this live-killed vaccine which has been adopted for commercial use in Japan.

In mainland China, effective vaccination has been achieved by using the BEFV G protein split from a semi-purified virus preparation by using non-ionic detergent [147]. The vaccine, which uses a white oil adjuvant, was shown to induce a neutralising antibody response and to protect 50% of cattle challenged 6 months after two subcutaneous doses at an interval of 3 weeks. All cattle with neutralising antibody titres >4 were found to be resistant to challenge. The vaccine has been adopted for field use in China prior to predicted epizootics and, although no formal field evaluation has been reported, it appears to be safe and effective [147]. A G protein sub-unit vaccine administered in Quil A adjuvant has also been developed in Australia [16]. Two doses of the vaccine administered on days 0 and 21 or three doses administered on days 0, 7 and 36 resulted in 100% protection against experimental challenge on day 104. It was also shown that three doses of 0.32 μg of the purified G protein were required for effective protection against challenge at 46 days [16]. There has been no field evaluation of this vaccine and it has not been adopted commercially.

Vaccination experiments have also been conducted using the BEFV G protein delivered in recombinant virus vectors. Vaccination with four doses of the Neethling strain of lumpy skin disease virus expressing the BEFV G protein at 0, 3, 6 and 12 weeks induced a specific neutralising antibody and cell-mediated immune responses but failed to protect cattle challenged with BEFV 10 weeks after the last dose [148]. In the same experiment, a commercial South African live-attenuated BEFV vaccine induced a stronger neutralisating antibody response but provided incomplete protection. Similarly, vaccination of cattle at days 0 and 21 with recombinant BEFV G protein expressed from the NYBH strain of vaccinia virus has been shown to induce specific neutralising antibodies but a protection experiment was inconclusive due to the poor potency of the challenge virus [13].

In summary, although experimental and commercial BEF vaccines have been developed in various formulations, there are few reports of the evaluation of their efficacy in the field. Protective immunity for most of the vaccines appears to be of limited duration and so their efficacy may be poor unless additional booster doses are administered at intervals of 6 months to 1 year. There remains a need for further research to provide a more informed evaluation of performance in the field and to evaluate slow-release and other advanced technologies that may reduce the required number of doses and extend the duration of protection.

### Control of cattle movements

As viraemia is brief (3–5 days) and occurs soon after infection, the risks associated with the movement of infected cattle pertains mostly to rapid transport across relatively short distances and a brief quarantine period in a vector-free area should be sufficient to eliminate the risk of introduction of BEFV with imported cattle [149]. However, recent phylogenetic evidence that BEFV strains of the East Asian lineage appear to be circulating in the Middle East [2, 8, 18, 32] suggest that the livestock trade has been responsible for the inter-continental transfer of the viruses, either in cattle or in vectors that may have accompanied them. Although BEF is not an OIE-listed disease, some countries require that live cattle or bovine semen to be imported are tested and shown to be free of BEFV-neutralising antibodies.

### Treatment

BEF is rare amongst viral diseases in that rationally based treatment is possible [54]. Rest, protection from the elements and the provision of feed and water will assist recovery. Laterally recumbent animals should be rolled-over several times a day to prevent loss of circulation and muscle damage. Force-feeding is not advisable because of the risk of aspiration pneumonia due to an impaired swallowing reflex [149]. Non-steroidal anti-inflammatory drugs are effective in preventing the onset of clinical signs when given daily during the incubation period and can induce rapid recovery when given after the onset of clinical disease [150]. Clinical signs of hypocalcaemia (ruminal stasis, paresis, loss of reflex) can be treated by subcutaneous or intravenous injection of calcium borogluconate. Convalescent animals should not be stressed or worked for several days after clinical signs subside to ensure biochemical functions have returned to normal [54].

## Conclusions

BEF is a disease for which the economic and social impacts are not always obvious and are frequently underestimated. Epizootics are now occurring more frequently in some parts of the world, there are increasing reports of alarmingly high case-fatality rates, and there is potential, under the influence of climate change and through the livestock trade, for spread of the disease to regions that are presently free. Although the epizootiology has been studied extensively in some regions, little is known of the distribution, prevalence and impacts over vast areas of Africa and Asia, relatively few virus isolates have been recovered and sequenced, and the specific vectors are not clearly defined anywhere in the world. This severely limits our ability to assess the relative importance and risk of spread by wind-borne dispersal of vectors and translocation through movement of livestock, and to assess the potential for establishment as an enzootic disease in Europe or the Americas through transmission by local vectors. Although a multitude of experimental and commercial vaccines have been developed, usage rates are often poor due to the irregular nature of epizootics and the need for multiple doses, and there are few published reports of the evaluation of vaccines under conditions in the field. The role of related viruses in the epizootiology of BEF is also unclear. This knowledge deficit provides a fertile field for future research.

## Additional files

10.1186/s13567-015-0262-4 **Sternal and lateral recumbency in cattle with clinical BEF**. Movie showing cattle displaying sternal and lateral during the clinical phase of BEFV infection.
10.1186/s13567-015-0262-4 **Isolate details and Genbank accession numbers of the BEFV sequences used for phylogenetic analysis.** Table providing isolation details (number/country/location/date) of viruses and Genbank accession numbers of L protein sequences that were used for the phylogenetic analysis displayed in Figure 4.
