# Supplementary material for: Epidemiology and control of bovine ephemeral fever
Source: Vet Res. 2015 Oct 28;46:124. doi: 10.1186/s13567-015-0262-4 (PMC4624662; doi:10.1186/s13567-015-0262-4)
Supplement: Supplementary file 2 — 10.1186/s13567-015-0262-4 Isolate details and Genbank accession numbers of the BEFV sequences used for phylogenetic analysis. Table providing isolation details (number/country/location/date) of viruses and Genbank accession numbers of L protein sequences that were used for the phylogenetic analysis displayed in Figure 4. [file 13567_2015_262_MOESM2_ESM.doc]

**Additional file 1 Isolate details and Genbank accession numbers of the BEFV sequences used for phylogenetic analysis.**

| **Clade** | **Isolate** | **Location** | **Genbank** |
| --- | --- | --- | --- |
| **Australia** | BEFV/D905/AU/Merriwagga/2010 | Australia | KF679460 |
|  | BEFV/E015/AU/Kemps_Creek/2010 | Australia | KF679469 |
|  | BEFV/D997/AU/OConnell/2010 | Australia | KF679465 |
|  | BEFV/E008/AU/Balrandal/2010 | Australia | KF679467 |
|  | BEFV/D901/AU/Kangaloon/2010 | Australia | KF679459 |
|  | BEFV/D909/AU/Cobaro/2010 | Australia | KF679463 |
|  | BEFV/BVLB6/AU/Berrimah/2012 | Australia | KF679476 |
|  | BEFV/EMAI66/AU/Gloucester/2011 | Australia | KF679482 |
|  | BEFV/D821/AU/Maitland/2010 | Australia | KF679454 |
|  | BEFV/D826/AU/Lightning_Ridge/2010 | Australia | KF679455 |
|  | BEFV/D853/AU/Henty/2010 | Australia | KF679458 |
|  | BEFV/V7203/AU/Berrimah/2008 | Australia | KF679461 |
|  | BEFV/V8363/AU/Beatrice_Hill/2010 | Australia | KF679462 |
|  | BEFV/V6330/AU/Beatrice_Hill/2004 | Australia | KF679472 |
|  | BEFV/CS1952/AU/Amberley/1992 | Australia | KF679452 |
|  | BEFV/CS1951/AU/Willowbank/1992 | Australia | KF679436 |
|  | BEFV/X760/AU/Camden/1991 | Australia | KF679474 |
|  | BEFV/CS1920/AU/Injune/1989 | Australia | KF679495 |
|  | BEFV/CS1921/AU/Injune/1989 | Australia | KF679428 |
|  | BEFV/CS1900/AU/Peachester/1988 | Australia | KF679449 |
|  | BEFV/CS1907/AU/Samford/1989 | Australia | KF679450 |
|  | BEFV/CS1913/AU/Oakey/1989 | Australia | KF679424 |
|  | BEFV/CS1910/AU/Samford/1989 | Australia | KF679493 |
|  | BEFV/CS1917/AU/Oakey/1989 | Australia | KF679426 |
|  | BEFV/CS1915/AU/Amberley/1989 | Australia | KF679425 |
|  | BEFV/CS1899/AU/Boonah/1988 | Australia | KF679448 |
|  | BEFV/v933/AU/Beatrice_Hill/1986 | Australia | KF679470 |
|  | BEFV/CS1622/AU/Peachester/1984 | Australia | KF679420 |
|  | BEFV/CS967/AU/Kairi/1981 | Australia | KF679443 |
|  | BEFV/DPP54/AU/Tortilla_Flats/1981 | Australia | KF679486 |
|  | BEFV/CS1647/AU/Peachester/1984 | Australia | AF058322 |
|  | BEFV/V496/AU/Aberdeen/1974 | Australia | KF679485 |
|  | BEFV/V633/AU/Kiama/1974 | Australia | KF679481 |
|  | BEFV/CS1931/AU/Etna_Creek/1973 | Australia | KF679488 |
|  | BEFV/CS1180/AU/Peachester/1982 | Australia | AF058321 |
|  | BEFV/CS1188/AU/Long_Pocket/1982 | Australia | KF679439 |
|  | BEFV/CS1820/AU/Peachester/1976 | Australia | KF679410 |
|  | BEFV/CS1821/AU/Amberley/1975 | Australia | KF679411 |
|  | BEFV/CS1925/AU/Tolga/1975 | Australia | KF679414 |
|  | BEFV/CS1819/AU/Wacol/1973 | Australia | KF679409 |
|  | BEFV/CS1903/AU/Etna_Creek/1970 | Australia | KF679419 |
|  | BEFV/CS1818/AU/Upper_Barron/1970 | Australia | KF679408 |
|  | BEFV/CS1869/AU/Etna_Creek/1970 | Australia | KF679421 |
|  | BEFV/CS1942/AU/Camp_Mountain/1971 | Australia | KF679435 |
|  | BEFV/CS1940/AU/Etna_Creek/1970 | Australia | KF679433 |
|  | BEFV/CS1937/AU/Wongabah/1971 | Australia | KF679478 |
|  | BEFV/CS1938/AU/Munna/1970 | Australia | KF679451 |
|  | BEFV/CS42/AU/Beatrice_Hill/1975 | Australia | AF058324 |
|  | BEFV/CS1933/AU/Etna_Creek/1973 | Australia | KF679416 |
|  | BEFV/BB7721/AU/Charters_Towers/1968 | Australia | KF679404 |
|  | BEFV/CS1867/AU/Etna_Creek/1970 | Australia | KF679412 |
|  | BEFV/V1956/AU/Sydney/1956 | Australia | KF679437 |
| **Middle East** | BEFV/CP77/TR/2008 | Turkey | GQ229452 |
|  | BEFV/ADYMN/TR/2012 | Turkey | KC788421 |
|  | BEFV/ISR01/IL/2001 | Israel | JN833631 |
|  | BEFV/ISR00/IL/2000 | Israel | JN833630 |
|  | BEFV/ISR04/IL/2004 | Israel | JN833632 |
|  | BEFV/ISR10_3/IL/2010 | Israel | JN833635 |
|  | BEFV/ISR10_1/IL/2010 | Israel | JN833633 |
| **East Asia** | BEFV/YHL/JP/Yamagichi/1966 | Japan | AB462028 |
|  | BEFV/EGY12/EG/2012 | Egypt | KJ729108 |
|  | BEFV/JB76H/CN/1976 | China | JX564640 |
|  | BEFV/TN1_84/TW/1984 | Taiwan | AY935239 |
|  | BEFV/Onna3/JP/Okinawa/1989 | Japan | AB462040 |
|  | BEFV/ON89_1/JP/Okinawa/1989 | Japan | AB462037 |
|  | BEFV/Hirado_6/JP/Nagasaki/1988 | Japan | AB462029 |
|  | BEFV/ON88_3/JP/Okinawa/1988 | Japan | AB462035 |
|  | BEFV/ON89_2/JP/Okinawa/1989 | Japan | AB462038 |
|  | BEFV/LYC11/CN/2011 | China | JX564638 |
|  | BEFV/Shandong/CN/2011 | China | JX234571 |
|  | BEFV/LS11/CN/2011 | China | JX564637 |
|  | BEFV/Henan1/CN/2012 | China | KM276084 |
|  | BEFV/CP3/TR/2012 | Turkey | KC470310 |
|  | BEFV/CU16/TR/2012 | Turkey | KC470313 |
|  | BEFV/TN1_96/TW/1996 | Taiwan | AY935240 |
|  | BEFV/TN88128/TW/1999 | Taiwan | AF208840 |
|  | BEFV/JT02L/CN/2002 | China | JX564639 |
|  | BEFV/ON3_E12/JP/2012 | Japan | AB985267 |
|  | BEFV/TN1_01/TW/2001 | Taiwan | AY935241 |
|  | BEFV/ON01_3/JP/Okinawa/2001 | Japan | AB462043 |
|  | BEFV/ON01_1/JP/Okinawa/2001 | Japan | AB462041 |
|  | BEFV/TN8_01/TW/2001 | Taiwan | AY954457 |
|  | BEFV/TN9_01/TW/2001 | Taiwan | AY954458 |
|  | BEFV/TN124/TW/2004 | Taiwan | AY818194 |
|  | BEFV/ON04_1/JP/Okinawa/2004 | Japan | AB462044 |
